# Clinical development of a blood biomarker using apolipoprotein-A2 isoforms for early detection of pancreatic cancer

**DOI:** 10.1007/s00535-023-02072-w

**Published:** 2024-01-23

**Authors:** Ayumi Kashiro, Michimoto Kobayashi, Takanori Oh, Mitsuko Miyamoto, Jun Atsumi, Kengo Nagashima, Keiko Takeuchi, Satoshi Nara, Susumu Hijioka, Chigusa Morizane, Shojiro Kikuchi, Shingo Kato, Ken Kato, Hiroki Ochiai, Daisuke Obata, Yuya Shizume, Hiroshi Konishi, Yumiko Nomura, Kotone Matsuyama, Cassie Xie, Christin Wong, Ying Huang, Giman Jung, Sudhir Srivastava, Hiromu Kutsumi, Kazufumi Honda

**Affiliations:** 1 Department of Bioregulation, Graduate School of Medicine, Nippon Medical School, 1-1-5 Sendagi, Bunkyo-Ku, Tokyo, 113-8602 Japan; 2 Institute for Advanced Medical Sciences, Nippon Medical School, 1-1-5 Sendagi, Bunkyo-Ku, Tokyo, 113-8602 Japan; 3 Toray Industries, Inc., 2-1-1 Muromachi Nihonbashi, Chuo-Ku, Tokyo, 103-8666 Japan; 4 Keio University Hospital, 35 Shinanomachi, Shinjuku-Ku, Tokyo, 160-8582 Japan; 5 Department of Hepatobiliary and Pancreatic Surgery, National Cancer Center Hospital, 5-1-1 Tsukiji, Chuo-Ku, Tokyo, 104-0045 Japan; 6 Department of Hepatobiliary and Pancreatic Oncology, National Cancer Center Hospital, 5-1-1 Tsukiji, Chuo-Ku, Tokyo, 104-0045 Japan; 7 Institute of Advanced Medical Sciences, Hyogo Medical University, 1-1 Mukogawa, Nishinomiya, Hyogo 663-8501 Japan; 8 Department of Clinical Cancer Genomics, Yokohama City University Hospital, 3-9 Fukuura, Kanazawa-Ku, Yokohama, Kanagawa 236-0004 Japan; 9 Department of Head and Neck Esophageal Medical Oncology, National Cancer Center Hospital, 5-1-1 Tsukiji, Chuo-Ku, Tokyo, 104-0045 Japan; 10 Department of Gastroenterological Surgery, National Cancer Center Hospital, 5-1-1 Tsukiji, Chuo-Ku, Tokyo, 104-0045 Japan; 11 Center for Clinical Research and Advanced Medicine, Shiga University of Medical Science, Tsukiwamachi Seta, Otsu, Shiga 520-2192 Japan; 12 Japan Cancer Society, 5-3-3 Tsukiji, Chuo-Ku, Tokyo, 104-0045 Japan; 13 Department of Health Policy and Management, Nippon Medical School, 1-1-5 Sendagi, Bunkyo-Ku, Tokyo, 113-8602 Japan; 14 Biostatistics, Bioinformatics and Epidemiology Program, Vaccine and Infectious Disease Division, Fred Hutchinson Cancer Center, Seattle, WA 98109-1024 USA; 15 Bio Tool Department (Toray Molecular Oncology Lab.), Toray International America Inc., Brisbane, CA 94005 USA; 16 Division of Cancer Prevention, National Cancer Institute, Rockville, MD 20850 USA; 17 National Cancer Institute Early Detection Research Network, Rockville, MD 20850 USA

**Keywords:** Blood biomarker, Apolipoprotein A2-isoform, Early detection of pancreatic cancer, Carbohydrate antigen 19-9 (CA19-9)

## Abstract

**Background:**

We have previously reported apolipoprotein A2-isoforms (apoA2-is) as candidate plasma biomarkers for early-stage pancreatic cancer. The aim of this study was the clinical development of apoA2-is.

**Methods:**

We established a new enzyme-linked immunosorbent sandwich assay for apoA2-is under the Japanese medical device Quality Management System requirements and performed in vitro diagnostic tests with prespecified end points using 2732 plasma samples. The clinical equivalence and significance of apoA2-is were compared with CA19-9.

**Results:**

The point estimate of the area under the curve to distinguish between pancreatic cancer (*n* = 106) and healthy controls (*n* = 106) was higher for apoA2-ATQ/AT [0.879, 95% confidence interval (CI): 0.832–0.925] than for CA19-9 (0.849, 95% CI 0.793–0.905) and achieved the primary end point. The cutoff apoA2-ATQ/AT of 59.5 μg/mL was defined based on a specificity of 95% in 2000 healthy samples, and the reliability of specificities was confirmed in two independent healthy cohorts as 95.3% (*n* = 106, 95% CI 89.4–98.0%) and 95.8% (*n* = 400, 95% CI 93.3–97.3%). The sensitivities of apoA2-ATQ/AT for detecting both stage I (47.4%) and I/II (50%) pancreatic cancers were higher than those of CA19-9 (36.8% and 46.7%, respectively). The combination of apoA2-ATQ/AT (cutoff, 59.5 μg/mL) and CA19-9 (37 U/mL) increased the sensitivity for pancreatic cancer to 87.7% compared with 69.8% for CA19-9 alone. The clinical performance of apoA2-is was blindly confirmed by the National Cancer Institute Early Detection Research Network.

**Conclusions:**

The clinical performance of ApoA2-ATQ/AT as a blood biomarker is equivalent to or better than that of CA19-9.

**Supplementary Information:**

The online version contains supplementary material available at 10.1007/s00535-023-02072-w.

## Background

To reduce the mortality of patients diagnosed with pancreatic cancer, there is an urgent need for non-invasive screening methods using circulating blood biomarkers for pancreatic cancer. Carbohydrate antigen 19-9 (CA19-9) is widely used as a serum biomarker for the detection of pancreatic cancer in clinical practice, clinical follow-up of the efficacy of chemotherapy, monitoring of tumor recurrence, and prediction of prognosis of pancreatic cancer patients [1, 2]. However, the sensitivity of CA19-9 is not sufficiently high to detect patients eligible for curative surgery, and approximately 5–10% of the individuals who develop pancreatic cancer lack the enzyme for synthesizing CA19-9 [3]. Due to these limitations, CA19-9 is not currently recommended for use as a biomarker for the early detection and screening of pancreatic cancer [4]. Consequently, for the early detection of pancreatic cancer, it is necessary to replace CA19-9 or supplement with biomarkers that are complementary to CA19-9.

We recently identified unique processing patterns at the C-terminal ends of circulating homodimers of apolipoprotein-A2 (apolipoprotein-A2 isoform, apoA2-i) in patients with pancreatic cancer and high-risk individuals (HRIs) [5]. Circulating apoA2-i comprises five isoforms, apoA2-ATQ/ATQ, apoA2-ATQ/AT, apoA2-AT/AT, apoA2-AT/A, and apoA2-A/A, which are named according to the C-terminal sequences of the dimers. The alterations of processing patterns, referred to as hyper- or hypo-processing patterns, at the C-terminal ends of the amino acids of apoA2 dimers are caused by the morbid exocrine function involved in the secretion of exopeptidases by the pancreas (Supplemental Fig. 1) [6, 7]. In a previous study, we showed that apoA2-ATQ/AT is significantly reduced in patients with pancreatic cancer because the alteration of pancreatic exocrine functions causes aberrant processing of apoA2 dimers. To easily measure the concentration of apoA2-i, we previously developed sandwich enzyme-linked immunosorbent assay (ELISA) kits for Research Use Only (RUO) [8] and demonstrated the clinical application of the ELISA RUO kit for apoA2-i in several clinical studies focusing on the early detection of pancreatic cancer and HRIs [8].

However, the apoA2-i ELISA RUO kit was not designed for in vitro diagnostics (IVD) applications in clinical practice. We established two new assays in which apoA2-ATQ and apoA2-AT are measured separately and regenerated a sandwich ELISA kit with a novel combination of specific antibodies of IVD grade that was produced under the Japanese medical device Quality Management System requirements (QMS). In the present study, the performance of the apoA2-i ELISA kit for IVD use to discriminate pancreatic cancer was compared with that of CA19-9 using predefined end points as described in “Materials and methods”.

## Materials and methods

### Sample collection

Plasma samples and clinical information of all diseased and healthy individuals were collected retrospectively and managed using the Platform for Evaluating Biomarkers of Cancer Early Detection (P-EBED), which was administered by the National Cancer Center Hospital (Tokyo, Japan), Hyogo Medical University Hospital (Mukogawa, Japan), Yokohama City University Hospital (Yokohama, Japan), and Kagoshima Prefectural Comprehensive Health Center (Kagoshima, Japan) using the same standard operating procedure, between April 2017 and May 2021. All participants agreed to sample collection and provided written informed consent.

### Study design

#### Enrollment criteria for plasma samples

Plasma samples were collected from patients with pancreatic, esophageal, gastric, liver, and colorectal cancers, intraductal papillary mucinous neoplasms (IPMNs), and chronic pancreatitis. Malignancies that received no treatment before sample collection were collected. Healthy plasma samples were collected by experimental pancreatic cancer screening conducted in Kagoshima Prefecture, Japan (UMIN000028015). Experimental pancreatic cancer screening was performed by enrolling an asymptomatic population aged 50 years, as well as individuals who would become older than 50 years of age between April 1 and March 31 of the following year.

#### Exclusion of plasma samples

To accurately define the cutoff for healthy persons, participants who claimed or originally identified themselves healthy but later were with a definitive cancer diagnosis or any pancreatic disease were excluded from the study.

Cancer patients with treatment before blood collection or with a history of other cancer were excluded. One patient with pancreatic cancer who could not be diagnosed definitively as having pancreatic cancer at the time of blood collection was excluded at the discretion of the pancreatic specialist.

**Fig. 1:**
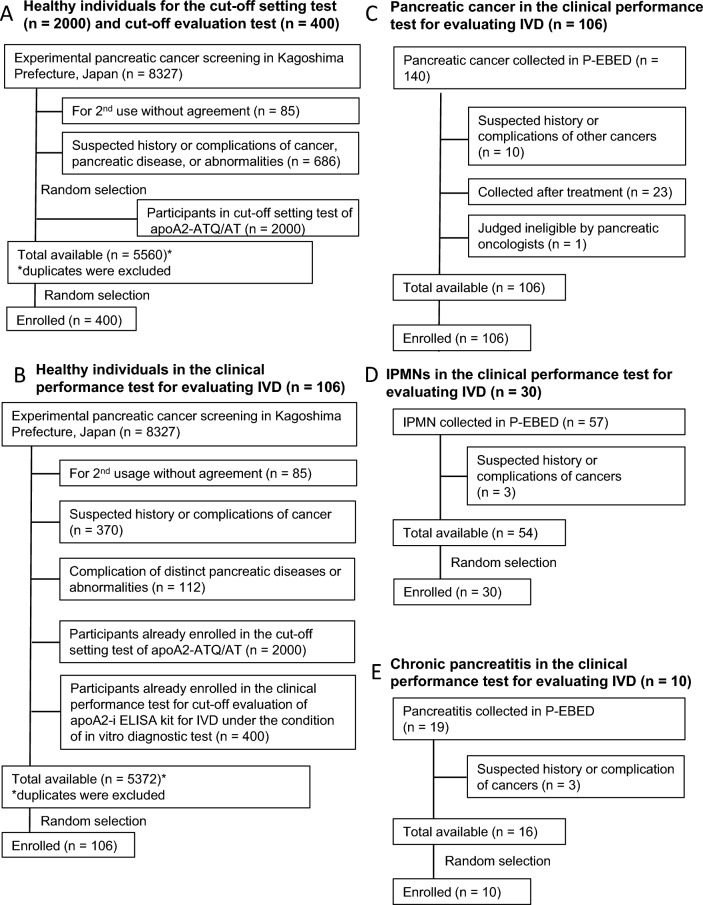
Selection criteria and eligibility requirements for enrollment of plasma samples in clinical investigations and the apoA2-i ELISA kit for the in vitro diagnostic (IVD) test. **A** Healthy individuals in the cutoff setting test (*n* = 2000) and in the cutoff evaluation test (*n* = 400). **B** Healthy individuals in the clinical performance test for evaluating the apoA2-i ELISA kit for IVD (*n* = 106). **C** Pancreatic cancer in the clinical performance test for evaluating the apoA2-i ELISA kit for IVD (*n* = 106). **D** IPMNs in the clinical performance test for evaluating the apoA2-i ELISA kit for IVD (*n* = 30). **E** Chronic pancreatitis in the clinical performance test for evaluating the apoA2-i ELISA kit for IVD (*n* = 10)

The healthy samples were selected separately from 8327 healthy individuals for cutoff setting, cutoff evaluation, and clinical performance evaluation testing so as not to duplicate each other. The details are described below and are shown in Fig. 1.

#### Cutoff setting for apoA2-ATQ/AT

From the plasma samples of 8327 healthy individuals, 2000 were randomly selected for testing. The selection criteria are shown in Fig. 1A. The cutoff for apoA2-ATQ/AT was defined as specificity of 95% in the 2000 individuals.

#### Clinical performance test for cutoff evaluation of the apoA2-i ELISA kit for IVD under in vitro diagnostic testing conditions

After excluding the 2000 of the 8327 asymptomatic participants who were the population for setting the cutoff, 400 healthy individuals were randomly selected. The eligibility criteria are shown in Fig. 1A.

#### Clinical performance evaluation of the apoA2-i ELISA kit for IVD to distinguish pancreatic cancer, chronic pancreatitis, and IPMNs compared to CA19-9 under IVD testing conditions

An additional healthy group of 106 individuals was randomly selected from the population of 8327 participants; the 2400 healthy individuals used in the two previous tests were excluded from this selection (Fig. 1B). Of the plasma samples collected from 140 patients with pancreatic cancer by P-EBED, samples from a total of 106 patients with pancreatic cancer were selected (Fig. 1C). The samples of 30 patients with IPMN (Fig. 1D) and 10 patients with chronic pancreatitis (Fig. 1E) were evaluated. Figure 1 shows the selection criteria for the plasma samples of healthy individuals, pancreatic cancer, chronic pancreatitis, and IPMNs.

#### Confirmatory test for cross-reactivity with other cancers

Samples from 30 patients with esophageal cancer, 30 patients with colorectal cancer, 10 patients with liver cancer, and 10 patients with gastric cancer were evaluated.

### Ethics approval

The present clinical study was approved by the ethics committee of Nippon Medical School (A-2020-064, A-2020-026, and A-2020-044), the central ethics committee of Nippon Medical School (M-2021-018), and the ethics review committee of Toray Industries, Inc. (HC2021-49, HC2021-54, HC2022-19, and HC2022-35).

### Configuration and measurement methods for IVD evaluation of the apoA2-i ELISA kit

The concentrations of apoA2-ATQ/ATQ and apoA2-AT/AT were defined using the apoA2-ATQ and apoA2-AT values, respectively, measured by IVD ELISA. Using these defined values, the concentration of apoA2-ATQ/AT was then calculated using the formula [(apoA2_ATQ/AT_) = $\sqrt {\left( {{\text{apoA}}2_{{{\text{ATQ}}}} {\text{*apoA}}2_{{{\text{AT}}}} } \right)}$], hereafter referred to as the APOA2-i Index. Participants with apoA2-AT or apoA2-ATQ concentrations below the lower limit of quantification (< 3.25 μg/mL and < 5.75 μg/mL, respectively) were defined as positive cases; i.e., these calculated values were set to 0 μg/mL. The apoA2-i ELISA kit for IVD is based on a two-step sandwich method and is designed to measure two types of apoA2-i (apoA2-AT and apoA2-ATQ) in plasma samples diluted up to 10,000-fold with sample diluent. The quantitative ranges for apoA2-AT and apoA2-ATQ are 0.325–20.8 ng/mL and 0.575–36.8 ng/mL, respectively.

A total of 100 µL of diluted plasma sample and standard solution were added to a pan-apoA2-antibody-coated, 96-well microplate and incubated at room temperature for 30 min. After washing, horseradish peroxidase (HRP)-conjugated-anti-apoA2-AT or HRP-conjugated-anti-apoA2-ATQ antibody was added for the detection of apoA2-AT or apoA2-ATQ, respectively, and the plates were incubated at room temperature for 30 min. After further washing, 100 µL of 3,3ʹ,5,5ʹ-tetramethylbenzidine solution were added as the substrate and incubated at room temperature for 30 min, and the absorbance of each well was measured at 450 and 650 nm as reference wavelengths. A four-parameter logistic curve generated from the absorbance values of the standard solutions was used to calculate the concentration of each apoA2-i in the sample.

### Measurement of CA19-9 and DUPAN2

CA19-9 concentration was measured using a chemiluminescent enzyme immunoassay kit for CA19-9 measurement (Lumipulse Presto CA19-9; FUJIREBIO Inc., Tokyo, Japan). CA19-9 concentrations of < 1.0 U/mL were treated as 1.0 U/mL, and those > 10,000 U/mL were treated as 10,000 U/mL. The cutoff value of CA19-9 was prespecified as 37.0 U/mL, as used in clinical settings. ApoA2-i and CA19-9 were measured by independent laboratories that were not associated with this study group. DUPAN2 was measured with plasma using DETERMINER DUPAN-2 N (Minaris Medical Co., Ltd., Tokyo, Japan). The cutoff value of DUPAN2 to diagnose pancreatic cancer was set > 150 U/mL.

### End points and achievement criterion for the clinical development of the apoA2-i IVD ELISA kit for IVD and clinical study

The primary and secondary end points, as well as the achievement standards, were predefined before starting measurements with the apoA2-i and CA19-9.

#### Primary end point

The primary end point was the area under the curve (AUC) on receiver-operating characteristic (ROC) curve analysis of apoA2-ATQ/AT to distinguish patients with pancreatic cancer from healthy individuals. The first achievement standard was a point estimation by AUC to distinguish individuals with pancreatic cancer from healthy individuals by calculating apoA2-ATQ/AT and CA19-9, defined as when the difference between the AUC values of apoA2-ATQ/AT and CA19-9 was greater than –0.05 [(AUC of apoA2-ATQ/AT)–(AUC of CA19-9) > –0.05].

#### Secondary end point

The concentration of apoA2-ATQ/AT was measured with the apoA2-i IVD ELISA using the 2000 plasma samples that were enrolled in the apoA2-ATQ/AT cutoff setting test, with the cutoff value of apoA2-ATQ/AT defined as specificity of 95%. The specificity of apoA2-ATQ/AT was then evaluated in the apoA2-ATQ/AT cutoff evaluation test. The achievement standard was specificity of apoA2-ATQ/AT confirmed as > 95% in the apoA2-ATQ/AT cutoff evaluation test. The sensitivities and 95% confidence intervals (95%CIs) of each of apoA2-ATQ/AT and CA19-9 were calculated in the clinical performance test for evaluating the apoA2-i IVD ELISA. The sensitivities and 95%CIs of stage I and II pancreatic cancers were calculated with apoA2-ATQ/AT and CA19-9, respectively, in the clinical performance test. The positive rate and 95%CI of apoA2-ATQ/AT were also calculated in individuals negative for CA19-9.

The specificity and positive rate of apoA2-ATQ/AT for chronic pancreatitis and IPMN were calculated. The positive predictive value (PPV) and negative predictive value (NPV) of each of apoA2-ATQ/AT and CA19-9 were calculated for incidences of pancreatic cancer ranging from 0.1 to 20%.

#### Additional analyses beyond the in vitro diagnostic test

The positive rates of apoA2-ATQ/AT for cancers other than pancreatic cancer were also calculated for the IVD test.

### Blinded confirmation of performance for early detection of pancreatic cancer by the National Cancer Institute Early Detection Research Network (NCI EDRN)

The positive and negative rates of the apoA2-i Index and CA19-9 were blindly confirmed by using the reference sets that were obtained from the NCI EDRN by the Toray Molecular Oncology Laboratory (Brisbane, CA). The data were analyzed by the EDRN Data Management and Coordination Center (DMCC). The reference set consisted of: healthy controls (*n* = 61), stage IA (*n* = 7); stage IB (*n* = 40); stage IIA (n = 8); stage IIB (*n* = 42), and stage II (-A/B unknown) (*n* = 1) of pancreatic cancer (total *n* = 98), acute benign biliary obstruction (*n* = 31), and chronic pancreatitis (*n* = 62). The pancreatic adenocarcinomas of EDRN were staged according to the criteria in the American Joint Committee on Cancer (AJCC) Staging Manual 7th edition.

### Statistical analysis

To determine the sample size for the clinical performance test for the evaluation of apoA2-ATQ/AT compared to CA19-9, it was necessary to control for the power of achieving the primary end point [(AUC of apoA2-ATQ/AT)–(AUC of CA19-9) > –0.05]. Simulations were conducted to calculate the probability of achieving the primary end point by performing 20,000 repeated samplings. Simulations assumed that apoA2-ATQ/AT and log-transformed CA19-9 followed a bivariate normal distribution with a correlation coefficient of 0, that both AUCs would be 0.80, and that the standard deviation (SD) in the pancreatic cancer patients would be twice that in healthy individuals. The probability of success was 86.9% when 107 patients with pancreatic cancer and 107 healthy individuals were included. To determine the sample size for the cutoff setting test, a precision-based approach was used to control for variability in the cutoff value (estimated value − true value). This sample size estimation used the apoA2-I data and an RUO reagent (Human APOA2 C-terminal ELISA kit; Toray Industries, Inc., Tokyo, Japan) from another study performed in Kagoshima Prefecture, Japan. The true cutoff value was the value of the 95% negative proportion for all 8327 participating healthy individuals. Simulations were conducted to calculate bias by performing 5000 repeated samplings in the 8327 healthy individuals. Bias fell within the range of approximately ± 1 μg/mL in 95% of replications when sampling 2000 healthy individuals. Therefore, bias was sufficiently controlled in 2000 healthy individuals. A precision-based approach to control the width of the 95%CI for specificity was used to determine the sample size for the cutoff validation. Assuming a specificity of 95% for apoA2-ATQ/AT, 400 healthy individuals enabled control of the width of the 95% CI to within ± 2.5% using Wilson’s score method.

For the primary end point, ROC analyses were performed separately for both apoA2-ATQ/AT and CA19-9 to estimate AUC and the difference, and two-sided 95%CIs were estimated using the Delong method. For each secondary end point, 95%CIs for binomial proportion were estimated using Wilson’s score method. PPV and NPV for detecting patients with pancreatic cancer for prevalences ranging from 0.1 to 20% were calculated using Bayes’ theorem. On post hoc analyses, subgroup analyses were performed by tumor size, and logistic regression analyses were conducted. Any missing data were treated as unknown, and no data were imputed. All statistical analyses were performed using SAS^®^ version 9.4 (SAS Institute Inc., Cary, NC, USA), Microsoft^®^ Excel^®^ for Microsoft 365 MSO version 2108 (Microsoft Corporation, Redmond, WA, USA), EZR [9], and GraphPad PRISM^®^ version 9 (MDF, Tokyo, Japan).

## Results

### Configuration and performance of the apoA2-i IVD ELISA

**Fig. 2:**
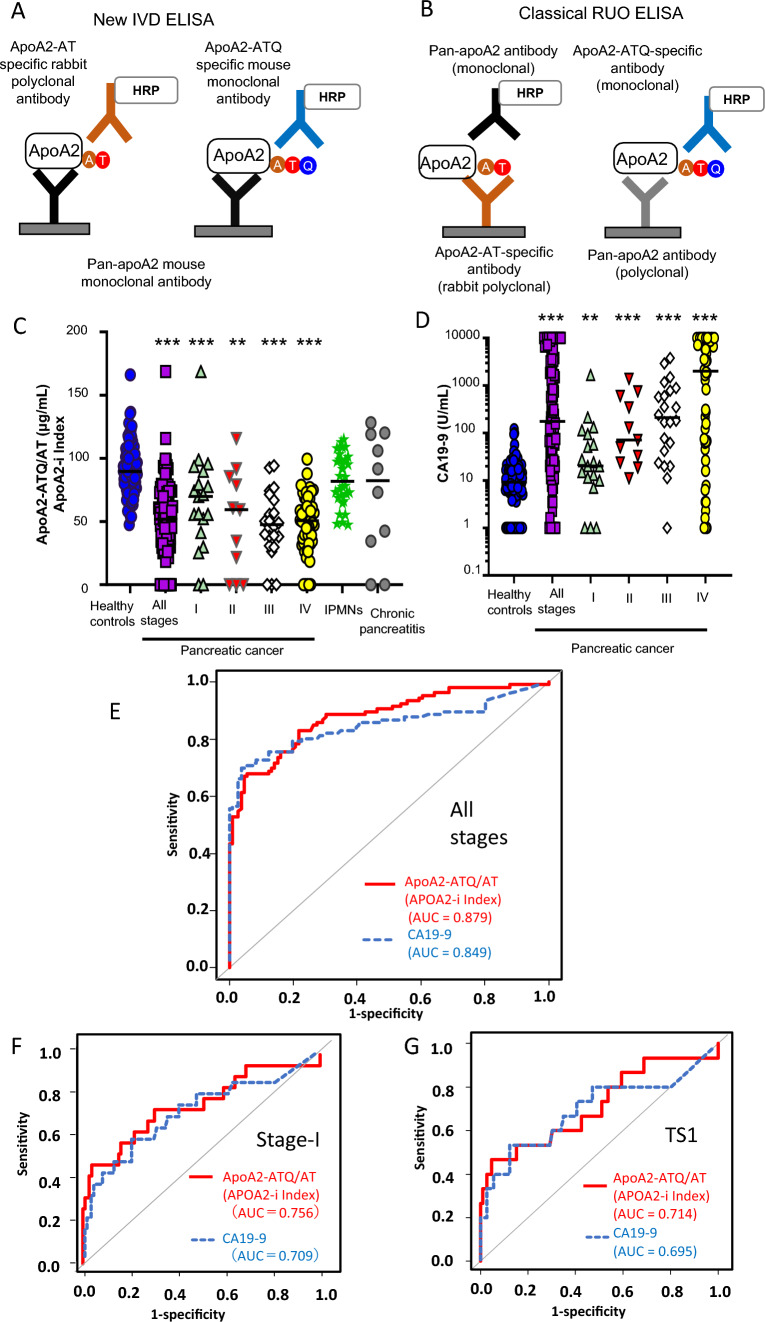
Configuration of the apoA2-i ELISA kit for research use only (RUO) and in vitro diagnostic (IVD) use (**A**, **B**). Distributions and discrimination performances of apoA2-i and CA19-9 in pancreatic cancer, high-risk individuals, and healthy controls (**C**–**G**). **A** The ELISA IVD configuration comprises two ELISAs for measuring apoA2-AT and apoA2-ATQ. Both assays for apoA2-AT and apoA2-ATQ use Pan-apoA2 monoclonal antibody as the solid phase antibody. The assay for apoA2-AT uses apoA2-AT-specific rabbit polyclonal antibody for the HRP-conjugated antibody. The assay for apoA2-ATQ uses ApoA2-ATQ-specific monoclonal antibody for the HRP-conjugated antibody. **B** The ELISA RUO kit comprises two ELISAs (classical type). The assay for apoA2-AT uses apoA2-AT-specific rabbit polyclonal antibody for the immobilized antibody and Pan-apoA2 monoclonal antibody for the HRP-conjugated antibody. The assay for apoA2-ATQ uses Pan-apoA2 polyclonal antibody for the immobilized antibody and ApoA2-ATQ-specific monoclonal antibody for the HRP-conjugated antibody. (C) Distribution of apoA2-ATQ/AT [APOA2-i Index; (apoA2_ATQ/AT_) = $\sqrt{({\text{apoA}}{2}_{{\text{ATQ}}}*{\text{apoA}}{2}_{{\text{AT}}})}$] in the plasma of healthy controls, each stage of pancreatic cancer, IPMNs, and chronic pancreatitis. Black bars: median values. The asterisks indicate significance on the Wilcoxon rank sum test compared to healthy controls (*p < 0.05; **p < 0.01; ***p < 0.001). **D** Distribution of CA19-9 levels in plasma of healthy controls and each stage of pancreatic cancer. Black bars: median values. The asterisks indicate significance on the Wilcoxon rank sum test compared to healthy controls (*p < 0.05; **p < 0.01; ***p < 0.001). **E** Primary end point: ROCs and AUCs of apoA2-ATQ/AT and CA19-9 (red line: apoA2-ATQ/AT, blue dashed line: CA19-9) between healthy controls and all stages of pancreatic cancer. **F** ROCs and AUCs of apoA2-ATQ/AT and CA19-9 (red line: apoA2-ATQ/AT, blue dashed line: CA19-9) between healthy controls and stage I pancreatic cancer. **G** ROCs and AUCs of apoA2-ATQ/AT and CA19-9 (red line: apoA2-ATQ/AT, blue dashed line: CA19-9) between healthy controls and TS1 (tumor size ≤ 2 cm) of pancreatic cancer

Differences in the configuration between the new IVD ELISA and the previous ELISA for RUO are shown in Fig. 2. IVD ELISA kits were produced under Japanese medical device QMS (Fig. 2A and B).

### Selective criteria for plasma samples obtained from participants and participants’ background clinical characteristics; discriminatory performance between pancreatic cancer and healthy controls as the primary end point; distribution of plasma apoA2-i concentration and discriminatory performance for stage I pancreatic cancer and pancreatic cancer < 2 cm

The criteria used for plasma samples obtained from participants are shown in Fig. 1 (details are provided in the Materials and methods section). The clinical characteristics of healthy controls and patients with pancreatic cancer, IPMN, and chronic pancreatitis are shown in Table 1. The concentration of plasma apoA2-ATQ/AT was significantly lower in individuals with any stage of pancreatic cancer than in healthy individuals (Fig. 2C). Figure 2D shows the distributions of CA19-9. The AUC of apoA2-ATQ/AT for distinguishing patients with pancreatic cancer from healthy individuals was 0.879 (95% CI 0.832–0.925), and the point estimate of AUC of apoA2-ATQ/AT was greater than 0.849 (95% CI 0.793–0.905) in CA19-9. The AUC obtained by subtracting CA19-9 from apoA2-ATQ/AT [AUC (apoA2-ATQ/AT)–AUC (CA19-9)] was + 0.029 (Fig. 2E). The primary end point that was predefined before starting measurement was achieved. The AUC values of apoA2-ATQ/AT and CA19-9 for detection of stage I were 0.756 (95% CI 0.616–0.895) and 0.709 (95% CI 0.559–0.860), respectively (Fig. 2F, Supplemental Table 1). The point estimate of AUCs of apoA2-ATQ/AT for stage I was greater than that of CA19-9. In addition, AUCs of apoA2-ATQ/AT and CA19-9 for detection of tumors < 2 cm (TS1) were 0.714 (95% CI 0.547–0.881) and 0.695 (0.517–0.873), respectively, and the AUC of apoA2-ATQ/AT was also greater than that of CA19-9 (Fig. 2G, Supplemental Table 1). The AUCs of apoA2-ATQ/AT and CA19-9 for detecting stage III and IV pancreatic cancers are shown in Supplemental Table 1.

**Table 1:** Clinical characteristics of plasma samples in the in vitro diagnostic test

	PDAC	Chronic pancreatitis	IPMN	Healthy
	(*N* = 106)	(*N* = 10)	(*N* = 30)	(*N* = 106)
Sex				
Male	60	7	19	54
Female	46	3	11	52
Age (y)				
*N*	106	10	30	106
Mean (SD)	65.2 (10.5)	62.8 (11.6)	66.7 (9.2)	63.2 (7.7)
Median	65	69	68	62.5
Min, Max	38, 86	47, 77	50, 85	50, 89
Age (y)				
< 65	51	4	12	61
≥ 65	55	6	18	45
Height (cm)				
*N*	106	0	3	88
Mean (SD)	161.45 (9.97)	–	170.20 (2.44)	160.51 (7.84)
Median	162.35	–	169.1	160.55
Min, Max	136.3, 183.2	–	168.5, 173.0	145.3, 178.0
Weight (kg)				
*N*	106	0	3	88
Mean (SD)	56.82 (11.34)	–	62.60 (1.22)	60.24 (10.69)
Median	56.05	–	62	60.3
Min, Max	34.5, 90.2	–	61.8, 64.0	36.5, 92.2
Stage^a^				
I	19	–	–	–
IA	8			
IB	11			
IIB	11	–	–	–
III	22	–	–	–
IV	54	–	–	–
TS1	15	–	–	–
Location				
Pancreatic head	40	–	–	–
Pancreatic body	30	–	–	–
Pancreatic body and tail	6	–	–	–
Pancreatic tail	17	–	–	–
Pancreatic head and body	1	–	–	–
Unknown	12	–	–	–

*PDAC* pancreatic ductal adenocarcinoma, *IPMN* intraductal papillary mucinous neoplasm, *SD* standard deviation, *TS1* tumor size ≤ 2.0 cm

^a^Union for International Cancer Control (UICC) classification 8th edition

### Definition and validity of the cutoff value of apoA2-ATQ/AT as secondary end points

**Fig. 3:**
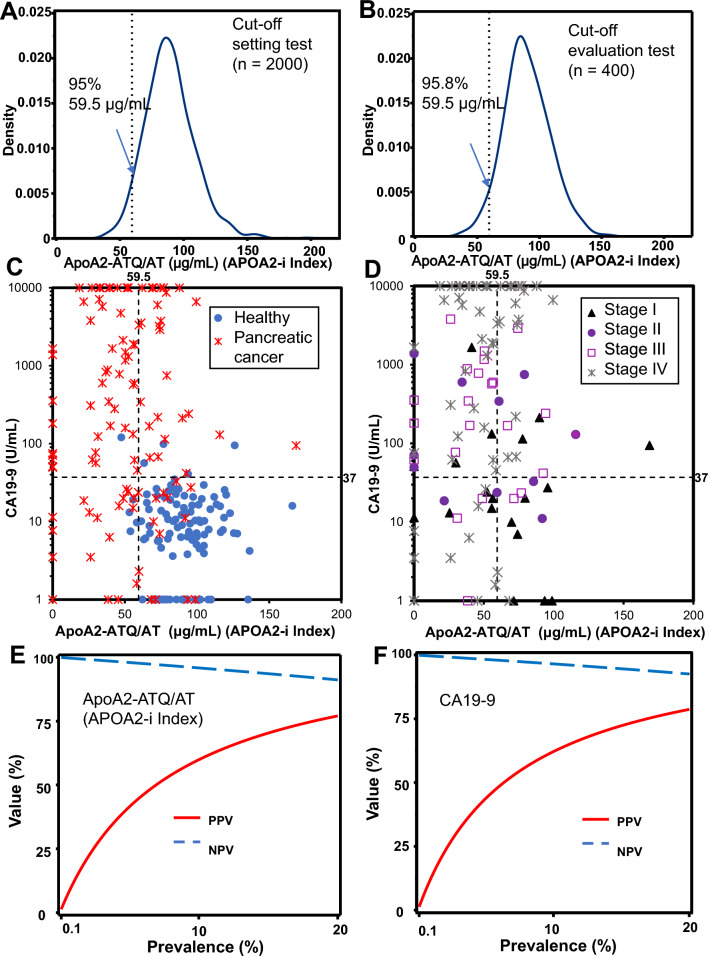
Validity of the cutoff value of apoA2-ATQ/AT, combination assay with apoA2-ATQ/AT and CA19-9, and positive predictive value (PPV) and negative predictive value (NPV) of apoA2-ATQ/AT and CA19-9. **A** Kernel density estimation of apoA2-ATQ/AT in the cutoff setting test. ApoA2-ATQ/AT with specificity of 95% is 59.5 μg/mL (dashed line: 95% specificity). **B** Kernel density estimation of apoA2-ATQ/AT in the cutoff estimation test. The specificity of apoA2-ATQ/AT of 59.5 μg/mL is 95.8% (dashed line: apoA2-ATQ/AT 59.5 μg/mL). Two-dimensional scatter plots of apoA2-ATQ/AT and CA19-9 in healthy controls and pancreatic cancer (**C**) (blue dots: healthy controls, red crosses: pancreatic cancer) and in each stage of pancreatic cancer (**D**) (black triangles: stage I, purple circles: stage II, purple squares: stage III, and gray crosses: stage IV pancreatic cancer) [black dashed lines: cutoff values of apoA2-ATQ/AT (59.5 μg/mL) and CA19-9 (37 U/mL)]. PPV and NPV of apoA2-ATQ/AT (**E**) and CA19-9 (**F**) in the population according to the prevalence of pancreatic cancer calculated by Bayesian estimation (red line: PPV, blue dashed line: NPV)

The frequency diagram in the cutoff setting test of apoA2-ATQ/AT that included 2000 healthy individuals (Table 2) showed a Gaussian distribution (Fig. 3A). The cutoff value of apoA2-ATQ/AT was identified as 59.5 μg/mL according to the predesigned protocols as the 95% point overall. The specificity for 400 healthy individuals (Table 2) of apoA2-ATQ/AT (≥ 59.5 μg/mL) was 95.8% (specificity 95% CI 93.3–97.3%). The reproducibility of the specificity of apoA2-ATQ/AT, the secondary end point, was achieved (details of secondary end points and the achievement criteria are provided in “Materials and methods”). A Gaussian distribution was also seen in 400 healthy individuals (Fig. 3B). Clinical performance tests of healthy controls (*n* = 106) confirmed the reliability of specificity as 95.3%. The frequencies of apoA2-AT and apoA2-ATQ are shown in Supplemental Fig. 2 and Supplemental Fig. 3, respectively.

**Table 2:** Clinical characteristics of healthy individuals in the cutoff selection and evaluation test

	Healthy individuals Cutoff setting test	Healthy individuals Clinical performance test for cutoff evaluation of the apoA2-i ELISA kit for IVD
Sex		
*N*	2000	400
Male	1025	205
Female	975	195
Age (y)		
*N*	2000	400
Mean (SD)	62.9 (8.0)	63.4 (8.6)
Median	63	63
Min, Max	49, 91	49, 94
Height (cm)		
*N*	1522	320
Mean (SD)	161.51 (8.60)	160.76 (8.75)
Median	161.4	160.35
Min, Max	134.0, 184.4	139.5, 180.3
Weight (kg)		
*N*	1522	320
Mean (SD)	61.38 (11.79)	60.40 (11.55)
Median	60.6	59.75
Min, Max	32.9, 120.0	36.7, 110.3
BS (mg/dL)		
*N*	1512	318
Mean (SD^1^)	102.6 (18.5)	102.7 (16.6)
Median	98	99
Min, Max	73, 313	78, 252
HbA1c (%)		
*N*	1505	314
Mean (SD)	5.60 (0.59)	5.65 (0.63)
Median	5.5	5.5
Min, Max	4.5, 11.2	4.4, 9.7
Amylase (U/L)		
*N*	1008	228
Mean (SD)	80.0 (29.6)	80.6 (32.1)
Median	74	75.5
Min, Max	26, 320	33, 359
HDL (mg/dL)		
*N*	1522	320
Mean (SD)	63.9 (16.7)	63.5 (15.5)
Median	62	62
Min, Max	25,126	33, 119
LDL (mg/dL)		
*N*	1522	320
Mean (SD)	127.5 (30.1)	128.4 (38.8)
Median	126	127
Min, Max	35, 256	43, 546

*SD* standard deviation, *BS* blood sugar, *HDL* high-density lipoprotein, *LDL* low-density lipoprotein

### Sensitivity for pancreatic cancer, positive rate for benign pancreatic disease, and the combined apoA2-ATQ/AT assay for negative CA19-9 in pancreatic cancer

When the cutoff values of apoA2-ATQ/AT and CA19-9 were set to 59.5 μg/mL and 37 U/mL, respectively, the sensitivities of apoA2-ATQ/AT and CA19-9 for detecting any stage of pancreatic cancer were 63.2% and 69.8%, respectively. In healthy individuals, specificity was 95.3% for both apoA2-ATQ/AT and CA19-9. The sensitivities of apoA2-ATQ/AT and CA19-9 for stage I pancreatic cancer were 47.4% and 36.8%, respectively, 50% and 46.7% for stage I/II, respectively, and 46.7% and 33.3% for TS1, respectively. The sensitivity of apoA2-ATQ/AT was thus higher than that of CA19-9 for the detection of stage I, stage I/II, and TS1 of pancreatic cancer (Table 3). The sensitivities of apoA2-ATQ/AT and CA19-9 for stage III and stage IV pancreatic cancers are shown in Table 3. The positive rates of apoA2-ATQ/AT for IPMN and chronic pancreatitis were 16.7% (95% CI 7.3–33.6%) and 40% (95% CI 16.8–68.7%), respectively (Supplemental Table 2).

**Table 3:** Sensitivities and specificities of the in vitro diagnostic test

			Sensitivity (%) [95%CI]		Sensitivity [95% CI]	Specificity (%) [95%CI]		Specificity [95%CI]
	Stage^a^	*N*	ApoA2-ATQ/AT (< 59.5 μg/mL^b^)	CA19-9 (> 37 U/mL^b^)	ApoA2-ATQ/AT (< 59.5 μg/mL^b^) CA19-9 (> 37 U/mL^b^)	ApoA2-ATQ/AT (< 59.5 μg/mL^b^)	CA19-9 (> 37 U/mL^b^)	ApoA2-ATQ/AT (< 59.5 μg/mL^b^) CA19-9 (> 37 U/mL^b^)
PDAC	All	106	63.2 [53.7, 71.8]	69.8 [60.5, 77.7]	87.7*** [80.1, 92.7]	–	–	–
	I	19	47.4 [27.3, 68.3]	36.8 [19.1, 59.0]	63.2* [41.0, 80.9]	–	–	–
	IA	8	37.5 [13.7, 69.4]	37.5 [13.7, 69.4]	62.5 [30.6, 86.3]	–	–	–
	IB	11	54.5 [28.0, 78.7]	36.4 [15.2, 64.6]	63.6 [35.4, 84.8]	–	–	–
	IIB	11	54.5 [28.0, 78.7]	63.6 [35.4, 84.8]	81.8 [52.3, 94.9]	–	–	–
	III	22	72.7 [51.8, 86.8]	77.3 [56.6, 89.9]	90.9 [72.2, 97.5]	–	–	–
	IV	54	66.7 [53.4, 77.8]	79.6 [67.1, 88.2]	96.3** [87.5, 99.0]	–	–	–
	I, II	30	50.0 [33.2, 66.8]	46.7 [30.2, 63.9]	70.0** [52.1, 83.3]	–	–	–
	TS1	15	46.7 [24.8, 69.9]	33.3 [15.2, 58.3]	66.7* [41.7, 84.8]	–	–	–
Healthy individuals		106	–	–	–	95.3 [89.4, 98.0]	95.3 [89.4, 98.0]	91.5 [84.6, 95.5]

*CI* confidence interval, *PDAC* pancreatic ductal adenocarcinoma, *TS1* tumor size ≤ 2.0 cm

^a^Stage: Union for International Cancer Control (UICC) classification 8th edition

^b^Cutoff value, significance of differences in sensitivity between combination of apoA2-ATQ/AT with CA19-9 and CA19-9 alone: *p < 0.05, **p < 0.01, and ***p < 0.001 (McNemar test)

Two-dimensional scatter plots of apoA2-ATQ/AT and CA19-9 in patients with pancreatic cancer and healthy individuals are shown in Fig. 3C. Of the 106 healthy individuals, 97 (91.5%, 95% CI 84.6–95.5%) were double-negative for apoA2-ATQ/AT (≥ 59.5 μg/mL) and CA19-9 (≤ 37 U/mL) (Fig. 3C). The sensitivity of the combination assay with apoA2-ATQ/AT and CA19-9 was 87.7%, which was significantly higher than the 69.8% for CA19-9 alone (Table 3). Figure 3D shows the distribution of apoA2-ATQ/AT and CA19-9 for each stage of pancreatic cancer. The sensitivities of the combination assay with apoA2-ATQ/AT and CA19-9 for stages I, IA, IB, IIB, I/II, and TS1 of pancreatic cancer were 63.2%, 62.5%, 63.6%, 81.8%, 70.0%, and 66.7%, respectively. In comparison, the sensitivities of CA19-9 alone for each stage were 36.8% (I), 37.5% (IA), 36.4% (IB), 63.6% (IIB), 46.7% (I/II), and 33.3% (TS1). These data and those obtained for other stages are shown in Table 3. The sensitivities of the combination assay with apoA2-ATQ/AT and CA19-9 to detect all stages, stage I, stage IV, stage I/II, and TS1 of pancreatic cancer were significantly higher than those of the CA19-9 assay alone (Table 3). The cross-tabulation results are shown in Supplemental Table 3. Although measurement of DUPAN2 had been not predefined before the design of this study, the results for DUPAN2 are shown in Supplemental Table 4 and Supplemental Fig. 4 as reference data.

### PPV and NPV for various prevalences of pancreatic cancer using Bayesian inference

PPV and NPV were calculated to identify patients in populations with various prevalences of pancreatic cancer (Fig. 3E, F). At prevalences of 0.1%, 1%, 10%, and 20%, the PPVs of apoA2-ATQ/AT were 1.3%, 12.0%, 59.9%, and 77.1%, respectively, and those of NPVs of apoA2-ATQ/AT were 100%, 99.6%, 95.9%, and 91.2%, respectively (Fig. 3E, Supplemental Table 5). The PPV and NPV of CA19-9 are shown in Fig. 3F and Supplemental Table 6.

### Cross-reactivity for other cancers

The reactivities of apoA2-ATQ/AT toward esophageal, gastric, colon, and liver cancer were 30% (9/30), 20% (2/10), 30% (9/30), and 20% (2/10), respectively (Supplemental Table 7).

### Logistic regression analyses of healthy individuals

To identify the clinical factors associated with apoA2-ATQ/AT, logistic regression analysis of samples from 2000 healthy individuals was performed (Table 4). On univariate analysis, significant odds ratios were identified for sex (male), age (≥ 65 years), hemoglobin A1c (≥ 6.5%), amylase (< 37 or > 125 U/L), and high-density lipoprotein (HDL) (< 40 mg/dL). On multivariate analysis, significant odds ratios were obtained for HbA1c, amylase, HDL, and low-density lipoprotein (LDL). HbA1c had the highest odds ratio in the multivariate analysis; the odds ratio of subjects with HbA1c ≥ 6.5% was 4.45 (95% CI 1.63–12.2%, *p* = 0.004) (Table 4).

**Table 4:** Subgroup analysis of specificity and odds ratios for the cutoff setting test (*N* = 2000)

	ApoA2-ATQ/AT negative frequency		Univariate analysis (*N* = 2000)		Multivariate analysis (*N* = 986)	
Factor		Specificity (%) [95% CI]	Crude odds ratio [95% CI]	*p* value	Adjusted odds ratio [95% CI]	*p* value
Male	962/1025	93.9 [92.2, 95.2]	1.66 [1.10, 2.52]	0.017	0.94 [0.46, 1.92]	0.865
Female	938/975	96.2 [94.8, 97.2]	1	–	1	–
Age < 65 y	1104/1152	95.8 [94.5, 96.8]	1	–	1	–
Age ≥ 65 y	796/848	93.9 [92.0, 95.3]	1.50 [1.00, 2.25]	0.047	0.82 [0.41, 1.63]	0.574
FBG < 100 mg/dL	792/823	96.2 [94.7, 97.3]	1	–	1	–
FBG ≥ 100 mg/dL	652/689	94.6 [92.7, 96.1]	1.45 [0.89, 2.36]	0.136	1.02 [0.48, 2.16]	0.952
HbA1c < 6.5%	1343/1402	95.8 [94.6, 96.7]	1	–	1	–
HbA1c ≥ 6.5%	93/103	90.3 [83.0, 94.6]	2.45 [1.21, 4.94]	0.013	4.45 [1.63, 12.20]	0.004
Amylase 37—125 U/L	903/935	96.6 [95.2, 97.6]	1	–	1	–
Amylase < 37 or > 125 U/L	67/73	91.8 [83.2, 96.2]	2.53 [1.02, 6.26]	0.045	2.82 [1.10, 7.23]	0.031
HDL < 40 mg/dL	53/67	79.1 [67.9, 87.1]	6.72 [3.52, 12.85]	< 0.001	3.75 [1.29, 10.92]	0.015
HDL ≥ 40 mg/dL	1400/1455	96.2 [95.1, 97.1]	1	–	1	–
LDL < 120 mg/dL	592/627	94.4 [92.3, 96.0]	1	–	1	–
LDL ≥ 120 mg/dL	861/895	96.2 [94.7, 97.3]	0.67 [0.41, 1.08]	0.102	0.35 [0.17, 0.73]	0.005

Missing data were excluded

Odds: number of positive cases/number of negative cases

*FBG* fasting blood glucose, *HDL* high-density lipoprotein, *LDL* low-density lipoprotein

### Blinded confirmation by the NCI EDRN

The point estimation of the AUC of the apoA2-i Index to distinguish stage I and II of pancreatic cancer from healthy controls was 0.836 (95% CI 0.774–0.898), higher than that of CA19-9 (0.783, 95% CI 0.710–0.855) (Supplemental Fig. 5). When the cutoff value of the apoA2-i Index was defined to be 54.47 (µg/mL) to achieve 95% specificity for healthy controls of the reference set of the NCI EDRN as the Japanese IVD test, the positive rates (sensitivities) of the apoA2-i Index for overall stage IA, IB, IIA, IIB, and overall stages were 57.1%, 55.0%, 37.5%, 69.0%, and 60.2%, respectively (Supplemental Table 8). Positive rates of CA19-9 using the cutoff of 37 U/mL were 28.6%, 55.0%, 50.0%, 57.1%, and 54.1%, respectively (Supplemental Table 8). Distributions of the concentration of the apoA2-i Index and CA19-9 are shown in Supplemental Fig. 6 and Supplemental Fig. 7. The estimated specificities for healthy controls of the apoA2-i Index and CA19-9 were both 95.1%.

## Discussion

This is the first study of the clinical performance of the new apoA2-i ELISA kit for IVD that meets Japanese medical device QMS and was produced to develop an in vitro diagnostic test for detecting pancreatic cancer.

Because apoA2 homodimers undergo a characteristic cleavage of C-terminal amino acids by pancreatic carboxypeptidase, each of the circulating apoA2 homodimers has a different C-terminal amino acid sequence. Pancreatic exocrine insufficiency and aberrant carboxypeptidase activity are often seen in patients with pancreatic cancer and in HRIs for pancreatic cancer [10, 11]. Therefore, it is considered that the predominance of apoA2-AT/AT (light isoform) or apoA2-ATQ/ATQ (heavy isoform) depends on the increase or decrease in exocrine function of the pancreas in patients with pancreatic cancer and HRIs. Patients who predominantly showed apoA2-AT/AT were referred to as the hyper-processing type, and those who predominantly showed apoA2-ATQ/ATQ were referred to as the hypo-processing type. In any case, apoA2-ATQ/AT (intermediate type) decreased in both hyper-processing and hypo-processing patterns due to the predominance of apoA2-ATQ/ATQ or apoA2-AT/AT (Supplemental Fig. 1).

In fact, after having been first reported by us, the reduction of specific homodimers of apoA2-i with C-terminal amino acid sequences has been confirmed in pancreatic cancer by several international research groups [12, 13]. Changes in the cleavage of C-terminal amino acid sequences are observed not only in pancreatic cancer, but also in autoimmune pancreatitis [14] and in radiation therapy for pancreatic cancer [15]. It is well known that exocrine dysfunction is seen in patients with autoimmune pancreatitis. In addition to destroying the pancreatic substrate, radiation therapy also leads to exocrine dysfunction. These observations do not contradict our hypothesis that the cleavage mechanism of apoA2-i is associated with the exocrine function of the pancreas.

Because CA19-9 is widely used in clinical practice, it is inevitable that the present study would have included patients whose discovery was triggered by high CA19-9 levels, and it is likely that there is bias in favor of CA19-9 compared to apoA2-i. Therefore, abnormal biomarker performance equivalence between CA19-9 and apoA2-i was defined as a difference in AUCs greater than − 0.05, calculated by the formula [(AUC of apoA2-ATQ/AT) – (AUC of CA19-9)]. However, the actual difference in AUC point estimates between apoA2-i and CA19-9 was + 0.029 (i.e., the point estimate was higher for apoA2-i than for CA19-9). These results are not only consistent within the present study, but also with previous studies using mass spectrometry [5, 16, 17] and the results of the previous RUO ELISA that cannot be used in clinical practice. In a previous meta-analysis using CA19-9 to identify patients with pancreatic cancer, the AUC for detecting pancreatic cancer was 0.84 [18]. The AUC of CA19-9 that was calculated from the meta-analysis did not contradict the results of the present study (AUC = 0.849).

In addition, when the cutoff for apoA2-ATQ/AT (apoA2-i Index) was defined as 59.5 μg/mL according to the prespecified criterion, the sensitivities of apoA2-ATQ/AT for stage I and TS1 of pancreatic cancer were 47.4% and 46.7%, respectively, and both of these sensitivities were higher than those of CA19-9. The specificities of apoA2-ATQ/AT and CA19-9 were the same (95.3%). It was concluded that the clinical performance of apoA2-ATQ/AT as an apoA2-i ELISA kit for IVD is at least comparable to, or even greater than, that of CA19-9, because the prespecified primary and secondary end points could be achieved in the present study.

Interestingly, apoA2-ATQ/AT increased the detection rate of pancreatic cancer in patients with pancreatic cancer who were not detected by CA19-9. These results suggest that using a combination assay of both biomarkers might improve the rate of early detection of pancreatic cancer in clinical practice.

ApoA2-i is a biomarker used on a different basis from that of general liquid biopsy using a marker such as CA19-9. Although it is typically considered that liquid biopsy involves assessment of biomaterials that were specifically leaked from tumor tissues, apoA2-i is not leaked from tumor tissue. It was considered that the alteration of the cleavage of C-terminal of apoA2 homodimers was induced by activation of carboxypeptidases, which were leaked and activated from the microenvironment reflected by pancreatic disorders. Because apoA2-i clearly reflects pancreatic exocrine function, it has the potential to enable detection of patients with stage I and II pancreatic cancers and HRIs such as IPMNs. On the other hand, apoA2-i was also reacting to advanced stages of pancreatic cancer, and sensitivities increased with the progression of the clinical stage. It was considered that the exocrine function of the pancreas would decrease in patients with advanced stages of pancreatic cancer.

The positive rates of CA19-9 for gastric, colorectal, and liver cancers have been shown to vary from 30 to 60% [7]. The cross-reactivities of apoA2-i were less than those of CA19-9. However, because the results of cross-reactivities were obtained from a small sample size, we should continue a validation study using massive real-world datasets obtained from the clinical setting with the apoA2-i IVD kit.

The US Prevention and Screening Task Force has given a grade of “recommendation D” to screening for pancreatic ductal adenocarcinoma (PDAC) in the general population, suggesting that not only is it not helpful, but there is potential for significant harm. Because the incidence of PDAC in the general population is low, at 12 per 100,000 population, it is considered that it will not be possible to establish an efficient program for pancreatic cancer screening [19]. In fact, Singhi et al. proposed that pancreatic cancer screening be restricted to the at-risk population of PDAC with cystic precursor lesions such as IPMNs and newly developed adult-onset diabetes mellitus (NOD), but not in the general population [20].

A high-risk group for sporadic PDAC has been identified as subjects > 50 years old who have NOD [21]. In a retrospective, population-based study of 2122 NOD subjects, Chari et al. first reported that 18 (0.85%) cases of pancreatic ductal adenocarcinoma were diagnosed within 3 years of meeting the criteria for NOD, resulting in a six to eightfold higher risk for PDAC than in the general population [22]. A recent study suggested that hyperglycemia precedes PDAC diagnosis by approximately 36 months, providing a possible window of opportunity for the early detection of pancreatic cancer in NOD patients [23]. In the present study, multivariate logistic regression analysis showed that HbA1c ≥ 6.5% had the highest odds ratio for positivity of apoA2-ATQ/AT. This result suggests that there is some association between diabetes mellitus and the blood concentration of apoA2-ATQ/AT. In the USA, the US Congress passed the Recalcitrant Cancer Act, and the National Institutes of Health (NIH) proposed a list of priorities for PDAC research, foremost among them being the study of the relationship between diabetes mellitus and developing screening strategies for PDAC [23]. In future studies, we consider that the causal relationship of NOD with the risk of pancreatic cancer and apoA2-ATQ/AT should be investigated in screening for PDAC in the NOD population using blood biomarkers.

IPMN is considered to be a precancerous lesion of PDAC, and IPMN can progress from IPMN low-grade dysplasia (LGD) to high-grade dysplasia (HGD) and, subsequently, invasive carcinoma [24–26]. The results of our previous study on IPMN HGD, which is considered to be a non-invasive cancer, indicated that apoA2-ATQ/AT (sensitivity 70.6%, specificity 96.7%) had sufficiently higher sensitivity than CA19-9 (sensitivity 14.5%, specificity 98.9%) for detecting patients with IPMN HGD [27]. The results of previous IPMN HGD studies are consistent with the present findings that showed that apoA2-ATQ/AT detected patients with early-stage pancreatic cancer, such as stage I, with greater sensitivity than CA19-9.

Moreover, the detection of high-grade pancreatic intraepithelial neoplasia (HG PanIN), which is considered carcinoma in situ, is very important for the improvement of the overall survival of patients with PDAC [26]. However, there is no efficient modality for detecting HG PanIN. Therefore, in the present study, the clinical performance for HG PanIN could not be evaluated because no samples were available. To accurately evaluate sensitivity for the early stage of pancreatic cancer, it will be necessary to continue clinical studies of the detection of early-stage pancreatic cancer, including HG PanIN.

In addition, the positive rate of apoA2-ATQ/AT for chronic pancreatitis was 40% in the present study. The subpopulation of patients with chronic pancreatitis could not be distinguished from PDAC by blood testing for apoA2-i. Previous studies of risk stratification of IPMN have shown that apoA2-ATQ/AT is a potential biomarker for risk stratification of IPMN with malignant potential. Patients with chronic pancreatitis are also at risk for pancreatic cancer, similar to those with IPMN. It may be necessary to follow-up patients with chronic pancreatitis who are positive for apoA2-ATQ/AT.

We are considering that the clinical performance of the RUO and IVD of apoA2-i is almost the same, because Sato et al. reported that the AUC value was 0.889 in the previous study, which was investigated in a cross-sectional study using data obtained from the experimental cancer screening using the RUO reagent of apoA2-i. This result was very close to the IVD AUC (0.879) obtained in the present study [28].

In the present and previous studies [6, 27–29], we suggested that the blood concentration of apoA2-ATQ/AT is associated with not only early-stage PDAC, but also IPMN with a high-risk phenotype for pancreatic cancer and diabetes mellitus. ApoA2-ATQ/AT has the potential for risk stratification of pancreatic cancer in screening a population with NOD, pancreatic cysts, and IPMN.

To prove this hypothesis, it will be necessary to perform measurements in large global prospective studies using IVD ELISA in clinical practice.

The present study has several limitations. Many patients with pancreatic diseases in this study were referred and came to the National Cancer Center Hospital. Many of these patients had symptoms and were diagnosed on imaging or were positive for other biomarkers, so the true clinical performance of apoA2-i cannot be identified based solely on these results.

In the IVD test in the present study, only 30 patients with stage I/II pancreatic cancer were investigated. However, though it was not a prespecified clinical study, the blinded test by the NCI EDRN using 98 plasma samples of stage I/II showed that sensitivities for detecting stage IA, stage IIB, and stage I/II of the apoA2-i Index were higher than those of CA19-9. In addition, the point estimate of the AUC of apoA2 was higher than that of CA19-9. The results of the IVD test were reproducible in the NCI EDRN blinded test.

### Supplementary Information

Below is the link to the electronic supplementary material.

Supplementary file1 (DOCX 32 KB)

Supplementary file2 (PPTX 430 KB)

## Abbreviations

ApoA2-is: Apolipoprotein-A2 isoforms
AUC: Area under the curve
CA19-9: Carbohydrate antigen 19-9
HRIs: High-risk individuals
ELISA: Enzyme-linked immunosorbent assay
HRP: Horseradish peroxidase
IPMN: Intraductal papillary mucinous neoplasm
IVD: In vitro diagnostics
NCI EDRN: National Cancer Institute Early Detection Research Network
NPV: Negative predictive value
PDAC: Pancreatic ductal adenocarcinoma
P-EBED: Platform for evaluating biomarkers of cancer early detection
PPV: Positive predictive value
RUO: Research use only
ROC: Receiver-operating characteristic
SD: Standard deviation
SOP: Standard operating procedure
UICC: The Union for International Cancer Control
