# Supplementary material for: Clinical development of a blood biomarker using apolipoprotein-A2 isoforms for early detection of pancreatic cancer
Source: J Gastroenterol. 2024 Jan 23;59(3):263–78. doi: 10.1007/s00535-023-02072-w (PMC10904523; doi:10.1007/s00535-023-02072-w)
Supplement: Supplementary file 1 — Supplementary file1 (DOCX 32 KB) [file 535_2023_2072_MOESM1_ESM.docx]

| **Supplemental Table 1. Area under the curve for distinguishing pancreatic cancer cases from healthy individuals** | | | |  |
| --- | --- | --- | --- | --- |
|  | Stage* | ApoA2-ATQ/AT | CA19-9 |  |
| AUC [95%CI] | ALL | 0.879 [0.832, 0.925] | 0.849 [0.793, 0.905] |  |
|  | I | 0.756 [0.616, 0.895] | 0.709 [0.559, 0.860] |  |
|  | IA | 0.627 [0.363, 0.892] | 0.656 [0.391, 0.921] |  |
|  | IB | 0.849 [0.726, 0.972] | 0.748 [0.571, 0.924] |  |
|  | IIB | 0.797 [0.620, 0.975] | 0.920 [0.840, 1.000] |  |
|  | III | 0.908 [0.832, 0.984] | 0.913 [0.825, 1.000] |  |
|  | IV | 0.926 [0.885, 0.967] | 0.858 [0.777, 0.939] |  |
|  | I, II | 0.771 [0.661, 0.881] | 0.787 [0.680, 0.894] |  |
|  | TS1** | 0.714 [0.547, 0.881] | 0.695 [0.517, 0.873] |  |
| CI: confidence interval, *Union for International Cancer Control (UICC) classification 8th edition, **TS1: tumor size ≤2.0 cm | | | |  |
|  |  |  |  |  |
|  |  |  |  |  |

| **Supplemental Table 2. Positive rates of apoA2-ATQ/AT for IPMN and chronic pancreatitis** | | | |
| --- | --- | --- | --- |
|  |  | Positive rate [95%CI] |  |
|  | IPMN | 16.7 [7.3, 33.6] |  |
|  | Chronic pancreatitis | 40.0 [16.8, 68.7] |  |
|  | IPMN: intraductal papillary mucinous neoplasm, CI: confidence interval | | |
|  |  |  |  |

| **Supplemental Table 3. Cross-tabulation table of judgment results for apoA2-ATQ/AT and CA19-9** | | | | |
| --- | --- | --- | --- | --- |
|  |  |  |  |  |
|  |  | CA19-9 | | |
|  | ApoA2-ATQ/AT | Negative | Positive | Total |
| Healthy individuals | Negative | 97 | 4 | 101 |
|  | Positive | 4 | 1 | 5 |
|  | Total | 101 | 5 | 106 |
|  |  |  |  |  |
| PDAC | Negative | 13 | 26 | 39 |
|  | Positive | 19 | 48 | 67 |
|  | Total | 32 | 74 | 106 |
|  |  |  |  |  |
| Stage I* | Negative | 7 | 3 | 10 |
|  | Positive | 5 | 4 | 9 |
|  | Total | 12 | 7 | 19 |
|  |  |  |  |  |
| Stage II* | Negative | 2 | 3 | 5 |
|  | Positive | 2 | 4 | 6 |
|  | Total | 4 | 7 | 11 |
|  |  |  |  |  |
| Stage III* | Negative | 2 | 4 | 6 |
|  | Positive | 3 | 13 | 16 |
|  | Total | 5 | 17 | 22 |
|  |  |  |  |  |
| Stage IV* | Negative | 2 | 16 | 18 |
|  | Positive | 9 | 27 | 36 |
|  | Total | 11 | 43 | 54 |
|  |  |  |  |  |
| Stage I.II* | Negative | 9 | 6 | 15 |
|  | Positive | 7 | 8 | 15 |
|  | Total | 16 | 14 | 30 |
|  |  |  |  |  |
| Tumor size ≤ 2.0 cm | Negative | 5 | 3 | 8 |
|  | Positive | 5 | 2 | 7 |
|  | Total | 10 | 5 | 15 |
|  |  |  |  |  |
| Tumor size > 2.0 and ≤ 4.0 cm | Negative | 7 | 15 | 22 |
|  | Positive | 10 | 31 | 41 |
|  | Total | 17 | 46 | 63 |
|  |  |  |  |  |
| Tumor size > 4.0 cm | Negative | 1 | 6 | 7 |
|  | Positive | 3 | 11 | 14 |
|  | Total | 4 | 17 | 21 |
|  |  |  |  |  |
| Tumor size unknown | Negative | 0 | 2 | 2 |
|  | Positive | 1 | 4 | 5 |
|  | Total | 1 | 6 | 7 |

PDAC: pancreatic ductal adenocarcinoma

*The Union for International Cancer Control (UICC) classification 8th edition

| **Supplemental table 4. Positive rates of DUPAN2, CA19-9, apoA2-ATQ/AT, and those combination for healthy controls and pancreatic cancer** | | | | | | |  |
| --- | --- | --- | --- | --- | --- | --- | --- |
|  | Cases (number) | DUPAN2 | CA19-9 | ApoA2-ATQ/AT | DUPAN2 + CA19-9 | CA19-9 +  ApoA2-ATQ/AT |  |
| Healthy controls | 106 | 4.7 | 4.7 | 4.7 | 9.4 | 8.5 |  |
| Pancreatic cancer | 106 | 57.5 | 69.8 | 63.2 | 84.0 | 87.7 |  |
| Stages* |  |  |  |  |  |  |  |
| IA | 8 | 25.0 | 37.5 | 37.5 | 50.0 | 62.5 |  |
| IB | 11 | 18.2 | 36.4 | 54.5 | 45.5 | 63.6 |  |
| IIB | 11 | 54.5 | 63.6 | 54.5 | 72.7 | 81.8 |  |
| III | 22 | 40.9 | 77.3 | 72.7 | 86.4 | 90.9 |  |
| IV | 54 | 77.8 | 79.6 | 66.7 | 98.1 | 96.3 |  |
| I | 19 | 21.1 | 36.8 | 47.4 | 47.4 | 63.2 |  |
| I+II | 30 | 33.3 | 46.7 | 50.0 | 56.7 | 70.0 |  |
| * Union for International Cancer Control (UICC) classification 8th edition, cut-off; DUPAN2 (150 U/mL), CA19-9 (37 U/mL), and apoA2-ATQ/AT (59.5 μg/mL). The concentrations of each biomarker were calculated to one decimal place by rounding. | | | | | | |  |
|  |  |  |  |  |  |  |  |

| **Supplemental Table 5. PPV and NPV of apoA2-ATQ/AT according to the prevalence of pancreatic cancer in the population** | | | | | |
| --- | --- | --- | --- | --- | --- |
|  | Prevalence | PPV | NPV |  |  |
|  | 0.1% | 1.3% | 100% |  |  |
|  | 1% | 12% | 99.6% |  |  |
|  | 10% | 59.9% | 95.9% |  |  |
|  | 20% | 77.1% | 91.2% |  |  |
|  |  | | |  |  |

PPV: positive predictive value, NPV: negative predictive value

| **Supplemental Table 6. PPV and NPV of CA19-9 according to the prevalence of pancreatic cancer in the population** | | | | |
| --- | --- | --- | --- | --- |
|  |  |  |  |  |
| Prevalence | PPV | NPV |  |  |
| 0.1% | 1.5% | 100% |  |  |
| 1% | 13% | 99.7% |  |  |
| 10% | 62.3% | 96.6% |  |  |
| 20% | 78.8% | 92.7% |  |  |
| PPV: positive predictive value, NPV: negative predictive value | | |  |  |

| **Supplemental Table 7. Positive rates of apoA2-ATQ/AT in other cancers** | | |
| --- | --- | --- |
|  |  |  |
|  |  | Positive rate [95%CI] |
|  | Esophageal cancer | 30 [16.7, 47.9] |
|  | Gastric cancer | 20 [5.7, 51.0] |
|  | Colon cancer | 30 [16.7, 47.9] |
|  | Liver cancer | 20 [5.7, 51.0] |
|  | CI: confidence interval | |

| **Supplemental Table 8. Blinded confirmation study for positive rates of apoA2-ATQ/AT and CA19-9 by NCI EDRN** | | | | |
| --- | --- | --- | --- | --- |
|  |  |  | Sensitivity (%) | |
| PDAC | Stage* | N | ApoA2-ATQ/AT (< 54.47μg/mL**) | CA19-9 (> 37 U/mL**) |
|  | All | 98 | 60.2 | 54.1 |
|  | IA | 7 | 57.1 | 28.6 |
|  | IB | 40 | 55.0 | 55.0 |
|  | IIA | 8 | 37.5 | 50.0 |
|  | IIB | 42 | 69.0 | 57.1 |
|  | II^***^ | 1 | 100 | 100 |
| Acute Benign Biliary Obstruction |  | 31 | 38.7 | 48.4 |
| Chronic Pancreatitis |  | 62 | 50.0 | 12.9 |
| Healthy individuals positive rates  (Specificities) |  | 61 | 4.9  (Specificity 95.1) | 4.9  (Specificity 95.1) |

PDAC: pancreatic ductal adenocarcinoma,

* American Joint Committee on Cancer (AJCC) Staging Manual 7th edition,

**cut-off value, *** unknown of stage-IIA or IIB.
