# Supplementary material for: Clinical development of a blood biomarker using apolipoprotein-A2 isoforms for early detection of pancreatic cancer
Source: J Gastroenterol. 2024 Jan 23;59(3):263–78. doi: 10.1007/s00535-023-02072-w (PMC10904523; doi:10.1007/s00535-023-02072-w)
Supplement: Supplementary file 2 — Supplementary file2 (PPTX 430 KB) [file 535_2023_2072_MOESM2_ESM.pptx]

## Slide 1
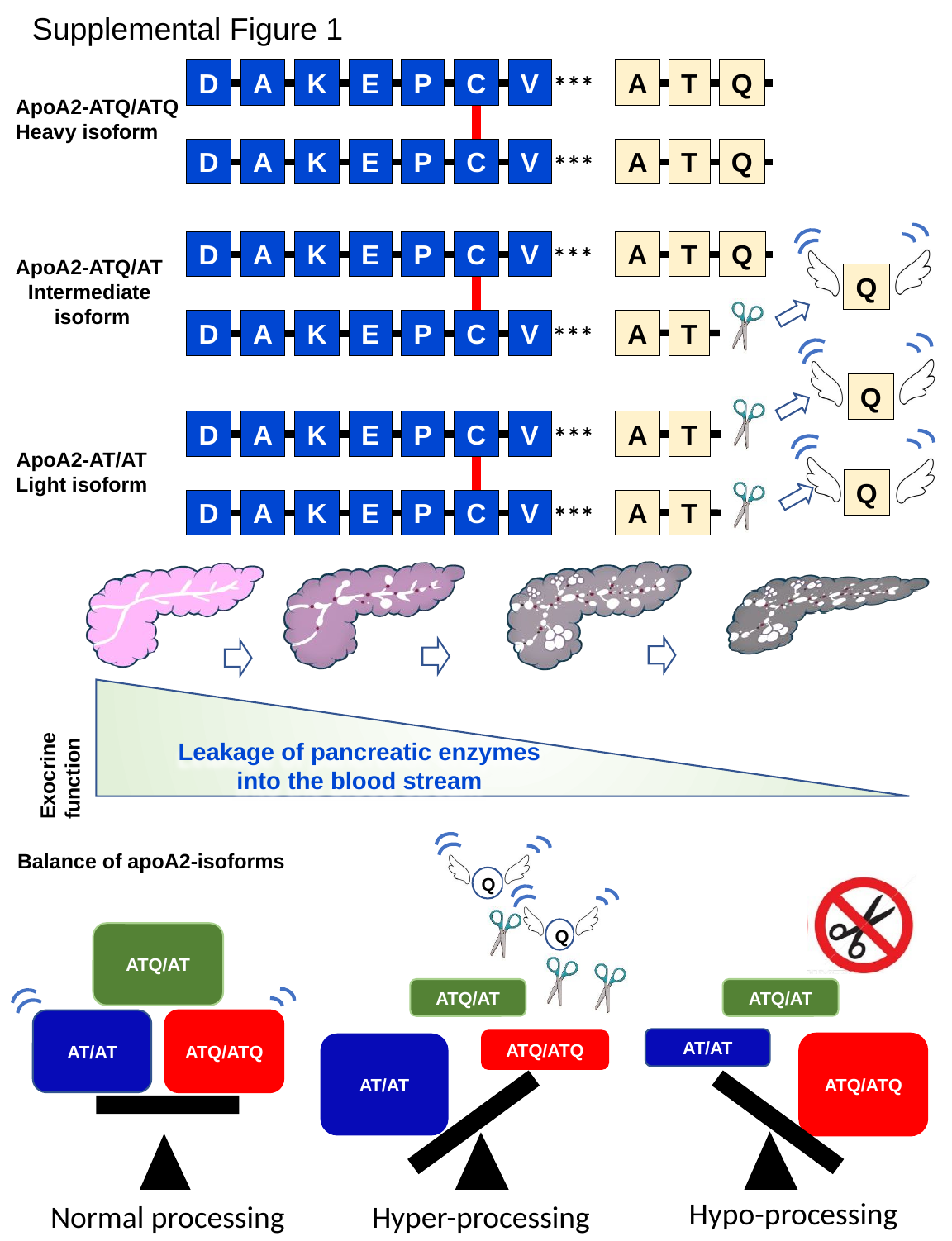

Supplemental Figure 1
D
A
K
E
P
C
V
A
T
Q
***
ApoA2-ATQ/ATQ
Heavy isoform
D
A
K
E
P
C
V
A
T
Q
***
D
A
K
E
P
C
V
A
T
Q
***
ApoA2-ATQ/AT
Intermediate
 isoform
Q
D
A
K
E
P
C
V
A
T
***
Q
D
A
K
E
P
C
V
A
T
***
Q
ApoA2-AT/AT
Light isoform
D
A
K
E
P
C
V
A
T
***
Exocrine
function
Leakage of pancreatic enzymes
into the blood stream
Q
Balance of apoA2-isoforms
Q
ATQ/AT
ATQ/AT
ATQ/AT
AT/AT
ATQ/ATQ
AT/AT
ATQ/ATQ
AT/AT
ATQ/ATQ
Hypo-processing
Normal processing
Hyper-processing

## Slide 2
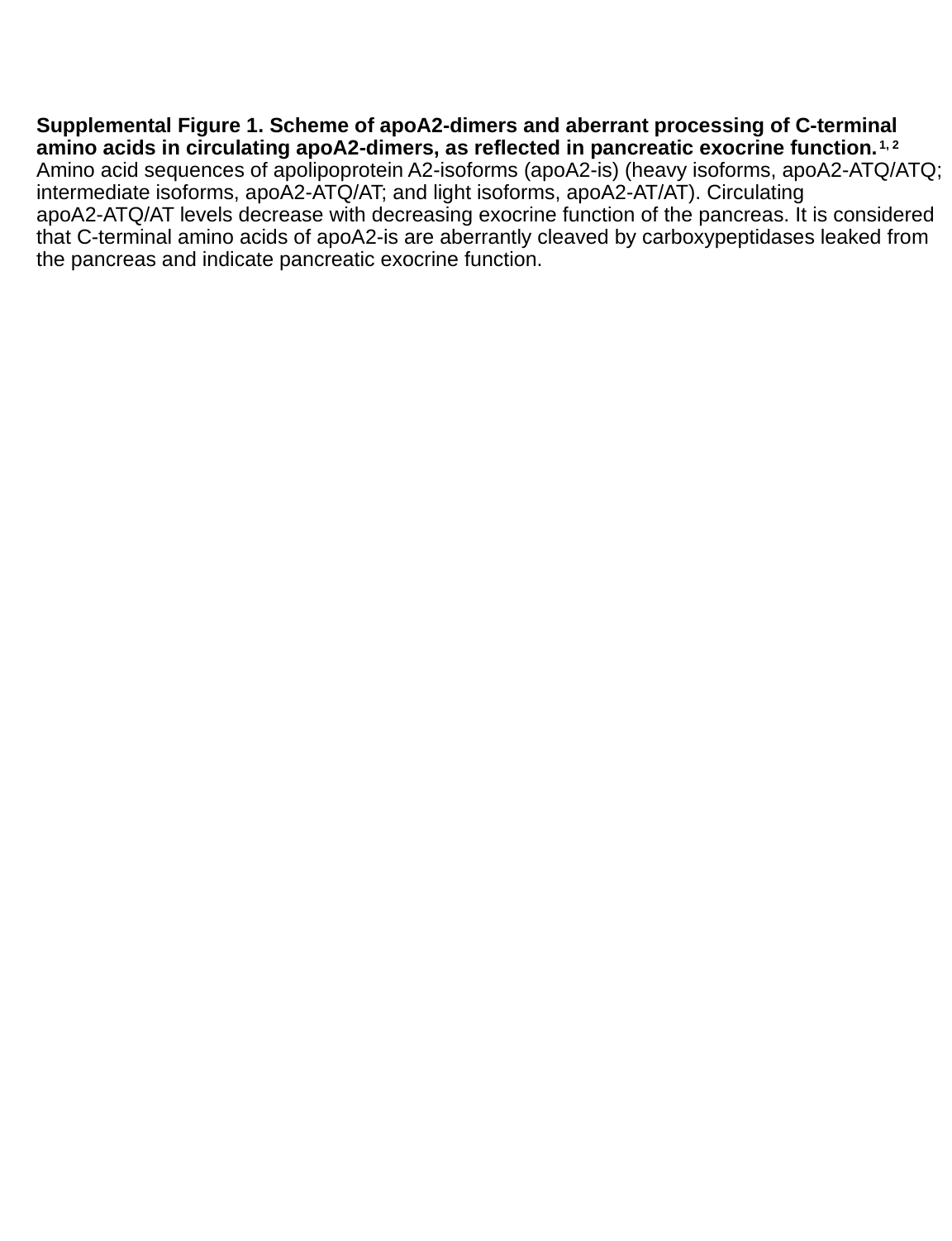

Supplemental Figure 1. Scheme of apoA2-dimers and aberrant processing of C-terminal amino acids in circulating apoA2-dimers, as reflected in pancreatic exocrine function.1, 2
Amino acid sequences of apolipoprotein A2-isoforms (apoA2-is) (heavy isoforms, apoA2-ATQ/ATQ; intermediate isoforms, apoA2-ATQ/AT; and light isoforms, apoA2-AT/AT). Circulating apoA2-ATQ/AT levels decrease with decreasing exocrine function of the pancreas. It is considered that C-terminal amino acids of apoA2-is are aberrantly cleaved by carboxypeptidases leaked from the pancreas and indicate pancreatic exocrine function.

## Slide 3
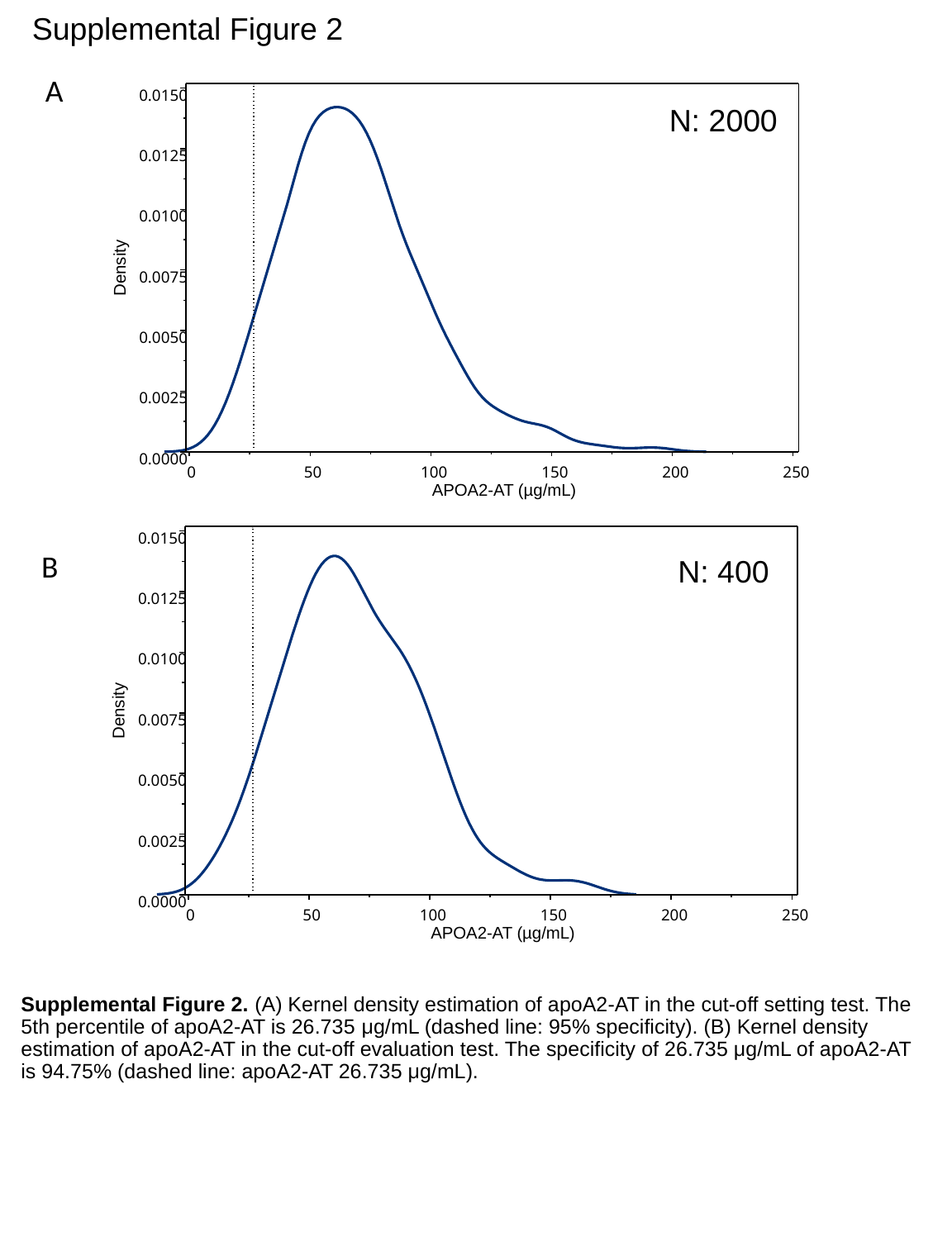

Supplemental Figure 2
A
0.0150
0.0125
0.0100
Density
0.0075
0.0050
0.0025
0.0000
0
50
100
150
200
250
APOA2-AT (µg/mL)
0.0150
0.0125
0.0100
Density
0.0075
0.0050
0.0025
0.0000
0
50
100
150
200
250
APOA2-AT (µg/mL)
N: 2000
B
N: 400
Supplemental Figure 2. (A) Kernel density estimation of apoA2-AT in the cut-off setting test. The 5th percentile of apoA2-AT is 26.735 μg/mL (dashed line: 95% specificity). (B) Kernel density estimation of apoA2-AT in the cut-off evaluation test. The specificity of 26.735 μg/mL of apoA2-AT is 94.75% (dashed line: apoA2-AT 26.735 μg/mL).

## Slide 4
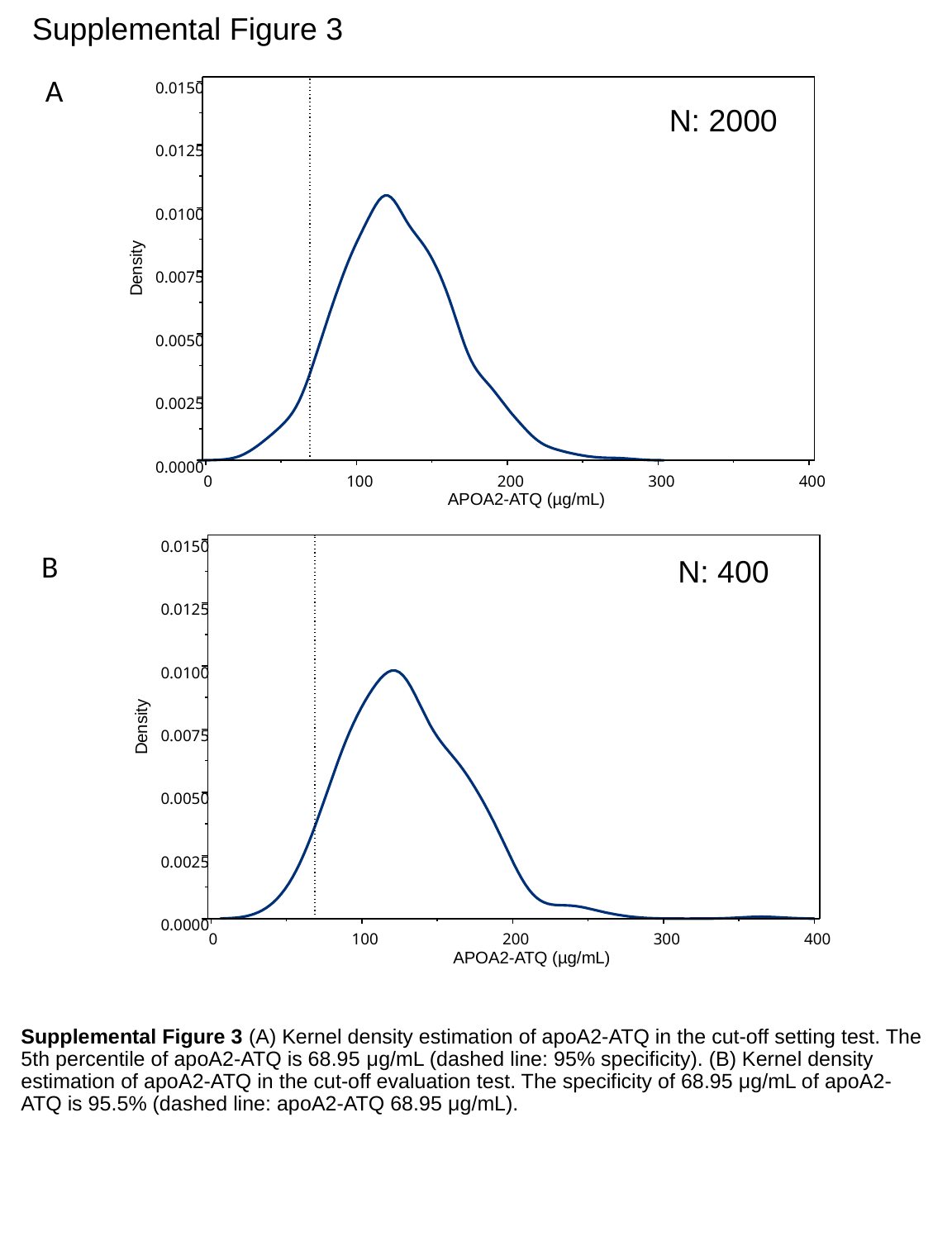

Supplemental Figure 3
A
0.0150
0.0125
0.0100
Density
0.0075
0.0050
0.0025
0.0000
0
100
200
300
400
APOA2-ATQ (µg/mL)
0.0150
0.0125
0.0100
Density
0.0075
0.0050
0.0025
0.0000
0
100
200
300
400
APOA2-ATQ (µg/mL)
N: 2000
B
N: 400
Supplemental Figure 3 (A) Kernel density estimation of apoA2-ATQ in the cut-off setting test. The 5th percentile of apoA2-ATQ is 68.95 μg/mL (dashed line: 95% specificity). (B) Kernel density estimation of apoA2-ATQ in the cut-off evaluation test. The specificity of 68.95 μg/mL of apoA2-ATQ is 95.5% (dashed line: apoA2-ATQ 68.95 μg/mL).

## Slide 5
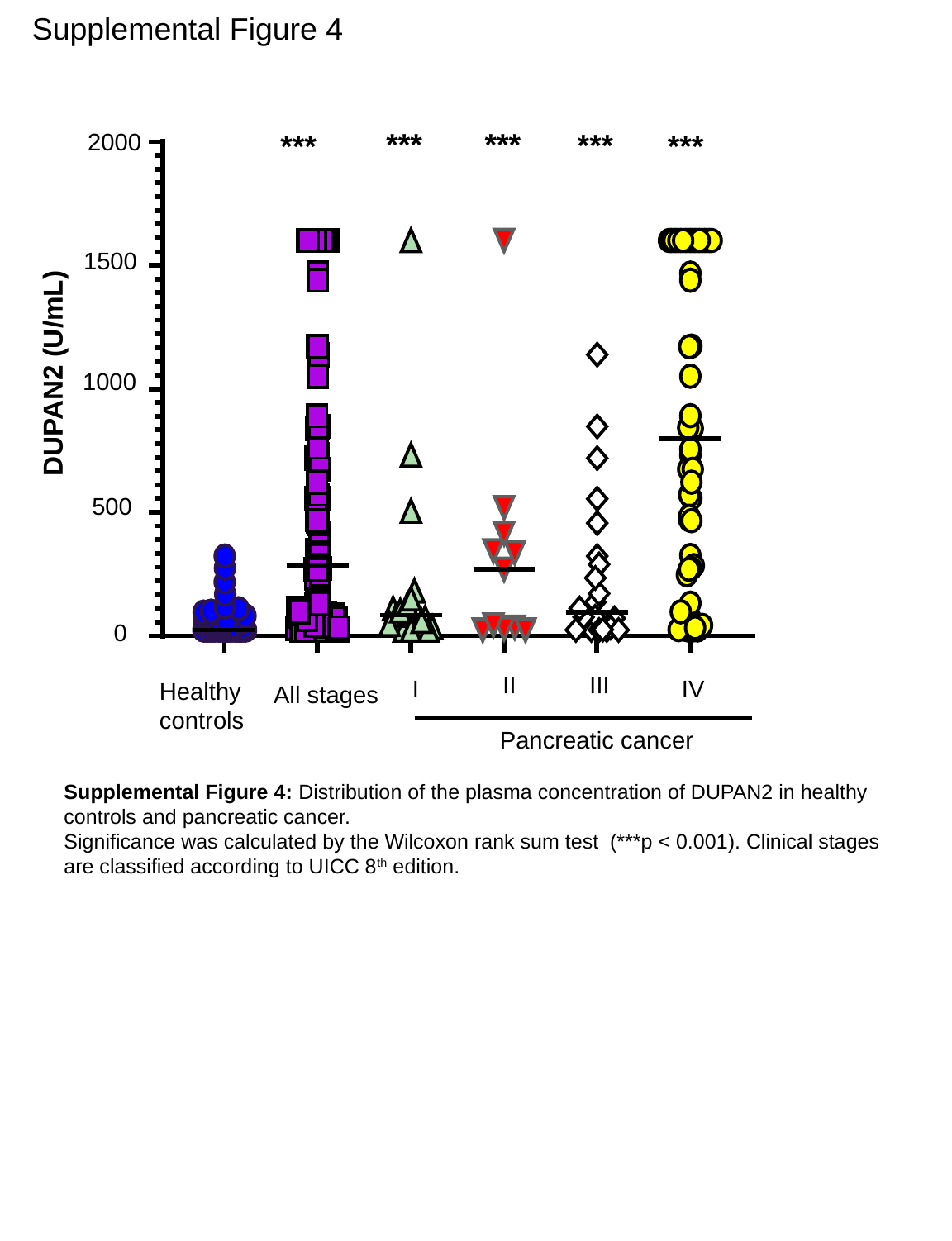

Supplemental Figure 4
***
***
***
***
***
2000
1500
DUPAN2 (U/mL)
1000
500
0
II
IV
I
Healthy controls
All stages
Pancreatic cancer
III
Supplemental Figure 4: Distribution of the plasma concentration of DUPAN2 in healthy controls and pancreatic cancer.
Significance was calculated by the Wilcoxon rank sum test (***p < 0.001). Clinical stages are classified according to UICC 8th edition.

## Slide 6
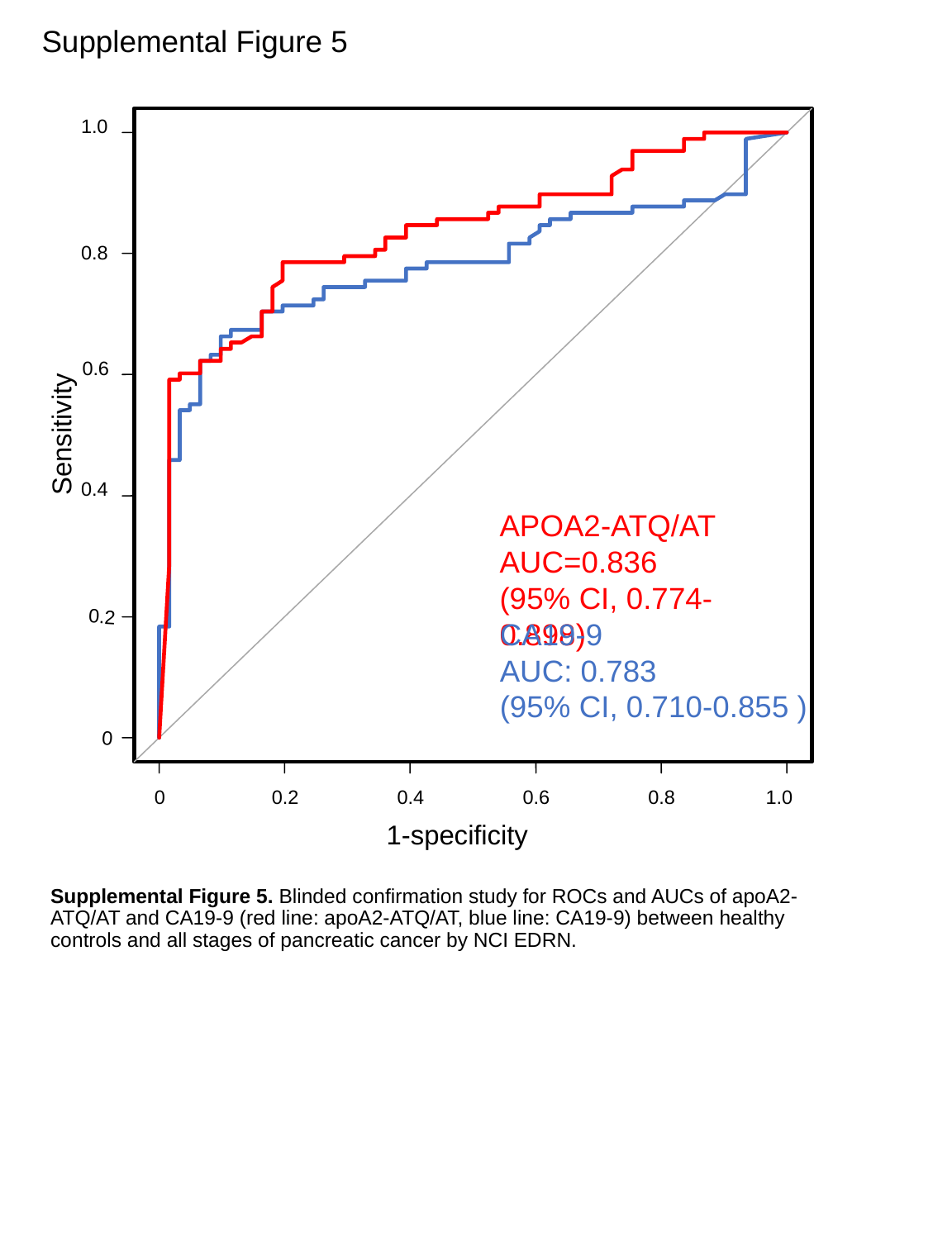

Supplemental Figure 5
1.0
0.8
 0.6
Sensitivity
0.4
APOA2-ATQ/AT
AUC=0.836
(95% CI, 0.774-0.898)
0.2
CA19-9
AUC: 0.783
(95% CI, 0.710-0.855 )
 0
 0
 0.2
 0.4
 0.6
 0.8
1.0
1-specificity
Supplemental Figure 5. Blinded confirmation study for ROCs and AUCs of apoA2-ATQ/AT and CA19-9 (red line: apoA2-ATQ/AT, blue line: CA19-9) between healthy controls and all stages of pancreatic cancer by NCI EDRN.

## Slide 7
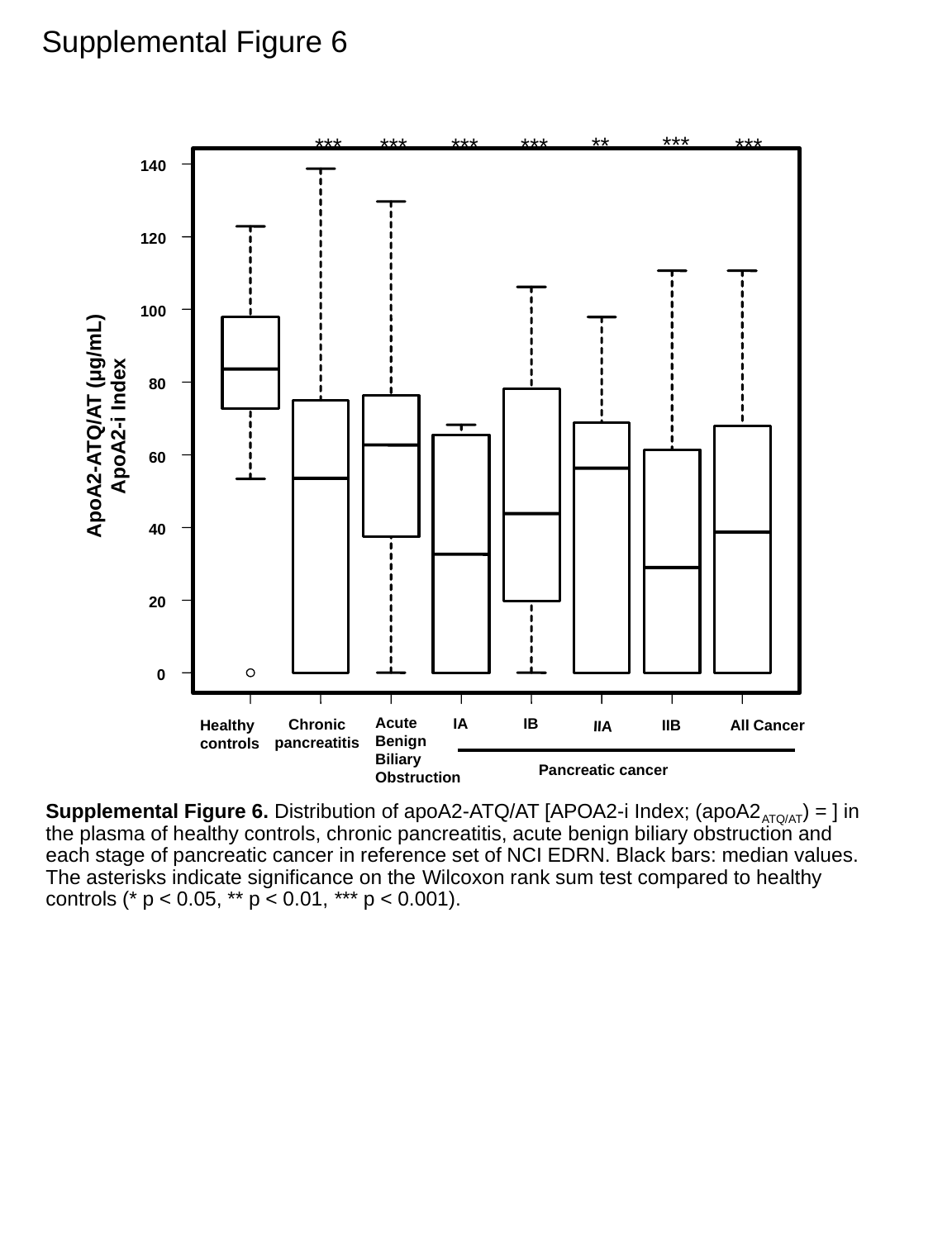

Supplemental Figure 6
***
**
***
***
***
***
***
140
120
100
80
ApoA2-ATQ/AT (μg/mL)
ApoA2-i Index
60
40
20
0
Chronic pancreatitis
Healthy controls
Acute
Benign
Biliary
Obstruction
IA
IB
IIB
 All Cancer
IIA
Pancreatic cancer

## Slide 8
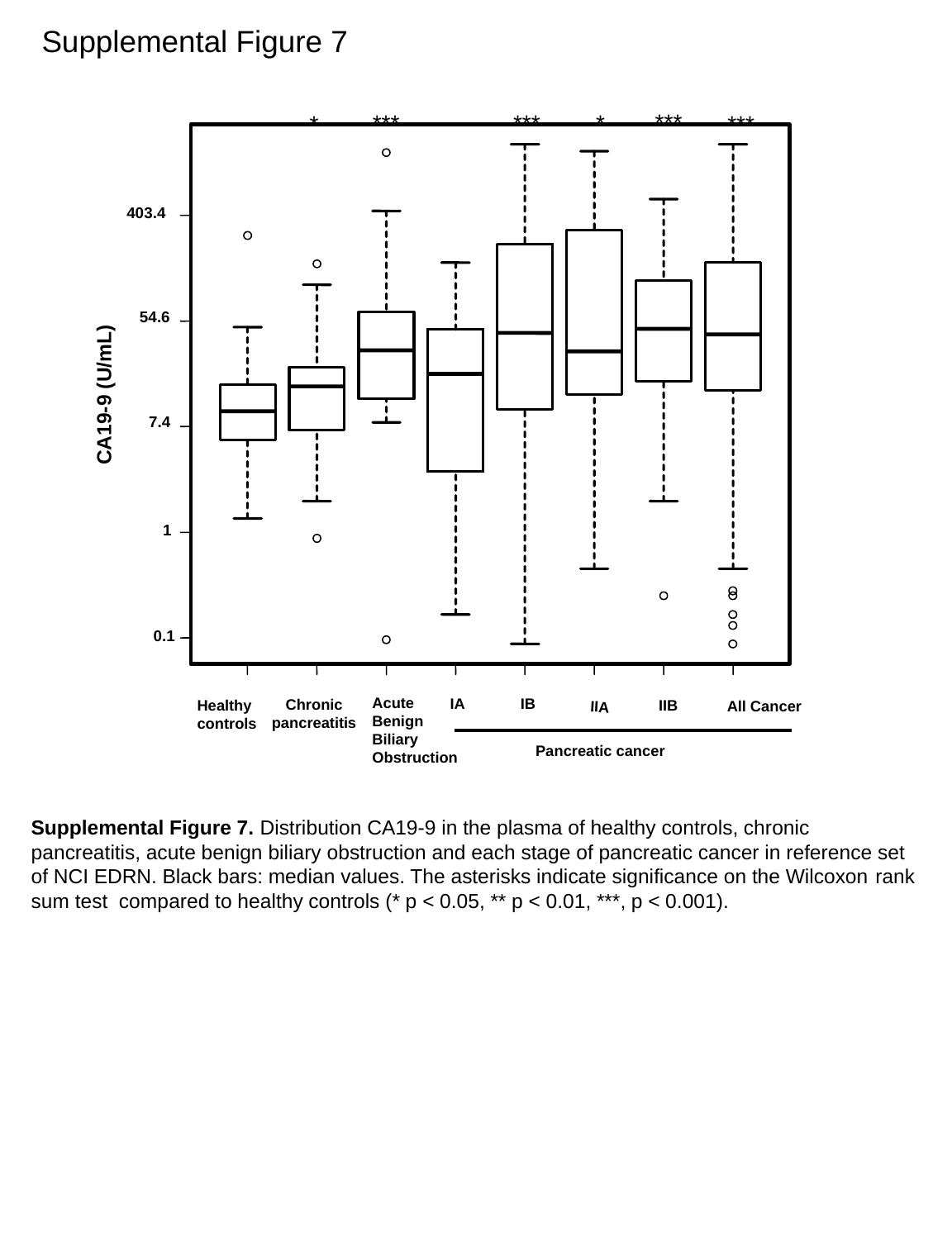

Supplemental Figure 7
***
*
***
***
***
*
403.4
54.6
7.4
1
0.1
CA19-9 (U/mL)
Chronic pancreatitis
Healthy controls
Acute
Benign
Biliary
Obstruction
IA
IB
IIB
 All Cancer
IIA
Pancreatic cancer
Supplemental Figure 7. Distribution CA19-9 in the plasma of healthy controls, chronic pancreatitis, acute benign biliary obstruction and each stage of pancreatic cancer in reference set of NCI EDRN. Black bars: median values. The asterisks indicate significance on the Wilcoxon rank sum test compared to healthy controls (* p < 0.05, ** p < 0.01, ***, p < 0.001).
